# Bile acid is a significant host factor shaping the gut microbiome of diet-induced obese mice

**DOI:** 10.1186/s12915-017-0462-7

**Published:** 2017-12-14

**Authors:** Xiaojiao Zheng, Fengjie Huang, Aihua Zhao, Sha Lei, Yunjing Zhang, Guoxiang Xie, Tianlu Chen, Chun Qu, Cynthia Rajani, Bing Dong, Defa Li, Wei Jia

**Affiliations:** 1 Shanghai Key Laboratory of Diabetes Mellitus and Center for Translational Medicine, Shanghai Jiao Tong University Affiliated Sixth People’s Hospital, 600 Yishan Rd, Shanghai, 200233 China; 2 University of Hawaii Cancer Center, 701 Ilalo St, Honolulu, HI 96813 USA; 3 National Key Laboratory of Animal Nutrition, China Agricultural University, 17 Qinghua East Rd, Beijing, 100193 China

**Keywords:** Bile acids, High-fat diet, Gut microbiota, Obesity, Microbe-metabolite interaction

## Abstract

**Background:**

Intestinal bacteria are known to regulate bile acid (BA) homeostasis via intestinal biotransformation of BAs and stimulation of the expression of fibroblast growth factor 19 through intestinal nuclear farnesoid X receptor (FXR). On the other hand, BAs directly regulate the gut microbiota with their strong antimicrobial activities. It remains unclear, however, how mammalian BAs cross-talk with gut microbiome and shape microbial composition in a dynamic and interactive way.

**Results:**

We quantitatively profiled small molecule metabolites derived from host-microbial co-metabolism in mice, demonstrating that BAs were the most significant factor correlated with microbial alterations among all types of endogenous metabolites. A high-fat diet (HFD) intervention resulted in a rapid and significant increase in the intestinal BA pool within 12 h, followed by an alteration in microbial composition at 24 h, providing supporting evidence that BAs are major dietary factors regulating gut microbiota. Feeding mice with BAs along with a normal diet induced an obese phenotype and obesity-associated gut microbial composition, similar to HFD-fed mice. Inhibition of hepatic BA biosynthesis under HFD conditions attenuated the HFD-induced gut microbiome alterations. Both inhibition of BAs and direct suppression of microbiota improved obese phenotypes.

**Conclusions:**

Our study highlights a liver–BA–gut microbiome metabolic axis that drives significant modifications of BA and microbiota compositions capable of triggering metabolic disorders, suggesting new therapeutic strategies targeting BA metabolism for metabolic diseases.

**Electronic supplementary material:**

The online version of this article (doi:10.1186/s12915-017-0462-7) contains supplementary material, which is available to authorized users.

## Background

There is growing concern that environmental factors, especially the ‘Western’ high-fat diet (HFD), have altered the genetic composition and metabolic activity of the mammalian gut microbiome [1, 2], predominantly through changes in the relative abundance of two dominant bacterial divisions, *Firmicutes* and *Bacteroidetes*. At the level of class and below, dramatic overgrowth of *Staphylococcus aureus* and *Enterobacteriaceae* (including *E. coli*) was observed in obese subjects [3]. The animal-based diet increased the abundance of *Alistipes*, *Bilophila*, and *Bacteroides* and decreased the levels of *Roseburia*, *Eubacterium rectale*, and *Ruminococcus bromii*, which metabolize dietary plant polysaccharides [1]. Recent studies have begun to focus on the effects of dietary interventions in reshaping the gut microbiota composition [1, 4]. However, much remains to be understood about designing and implementing treatments that are effective and adaptive in the mammalian gastrointestinal tract ecosystem. The gut microbiota composition is partially modulated by the extracellular metabolites that are derived from host and modified by microbes. Of these, bile acids (BAs) represent a highly abundant pool of host-derived and microbial-modified metabolites that are major regulators of the gut microbiome [5].

HFD-induced BA secretion as a driving force in shaping the obesity-associated gut microbial composition was first proposed in 2011 by Islam et al. [5], who observed that rats fed cholic acid (CA) experienced increases in *Firmicutes* accompanied by decreases in *Bacteroidetes* phyla; the resulting altered microbial signature was similar to that of obesity-associated gut microbiome. BAs are major constituents of bile, produced in the liver and then secreted into the duodenum to facilitate fat digestion and absorption [6]. They are antibacterial and create strong selective forces for the intestinal microbiota [1, 7]. Even within a single bacterial species, there can be differential sensitivity to specific BAs [8, 9]. Diet fat content can regulate the time and amount of secreted bile [10] and thus shape the microbiota. The impact of BAs on gut microbial composition has also been highlighted in murine colitis models [7]. Feeding mice a diet rich in milk-derived fat induced elevated levels of the BA taurocholic acid (TCA), facilitating the outgrowth of *Bilophila wadsworthia*, a bacteria species linked to the induction of colitis. Unfortunately, it remains unclear how the mammalian gut microbiome responds to short or long-term diet-induced changes in the BA pool. A systems-level understanding of microbe–microbe and microbe–metabolite interactions in the human gut is critical for understanding the potential limitations and opportunities of probiotic treatments. Probiotic treatments could potentially be useful in sequestering carcinogenic BAs and enriching those that exert beneficial effects on glucose and lipid metabolism in the host.

In the current study, we systematically evaluated the role of dietary fat-induced BA changes in shaping the gut microbial composition in male C57BL/6 mice. A global metabolome profiling of host–microbial co-metabolism implied BAs as a significant dietary factor that influenced changes in gut microbiota. HFD or BAs were administrated to mice and subsequent changes in both the BA pool and microbial compositions were profiled. Time-dependent profiles of gut microbiome responses to HFD were then monitored to determine the temporal relationship between BAs and alterations in the intestinal microbial composition. Additionally, microbiota changes were measured in response to the inhibition of hepatic BA biosynthesis. Our overall purpose was to establish a linkage between dietary fat, BAs, and specific alterations of the gut microbiome, which, in turn, would be capable of triggering metabolic disease. Identification of specific microbiota alterations in response to BAs may suggest new therapeutic strategies that target BA metabolism for the prevention and treatment of metabolic diseases.

## Results

### Correlation of metabolome with gut microbiome in response to HFD treatment revealed that BAs were an important dietary factor impacting gut microbiota composition

**Fig. 1:**
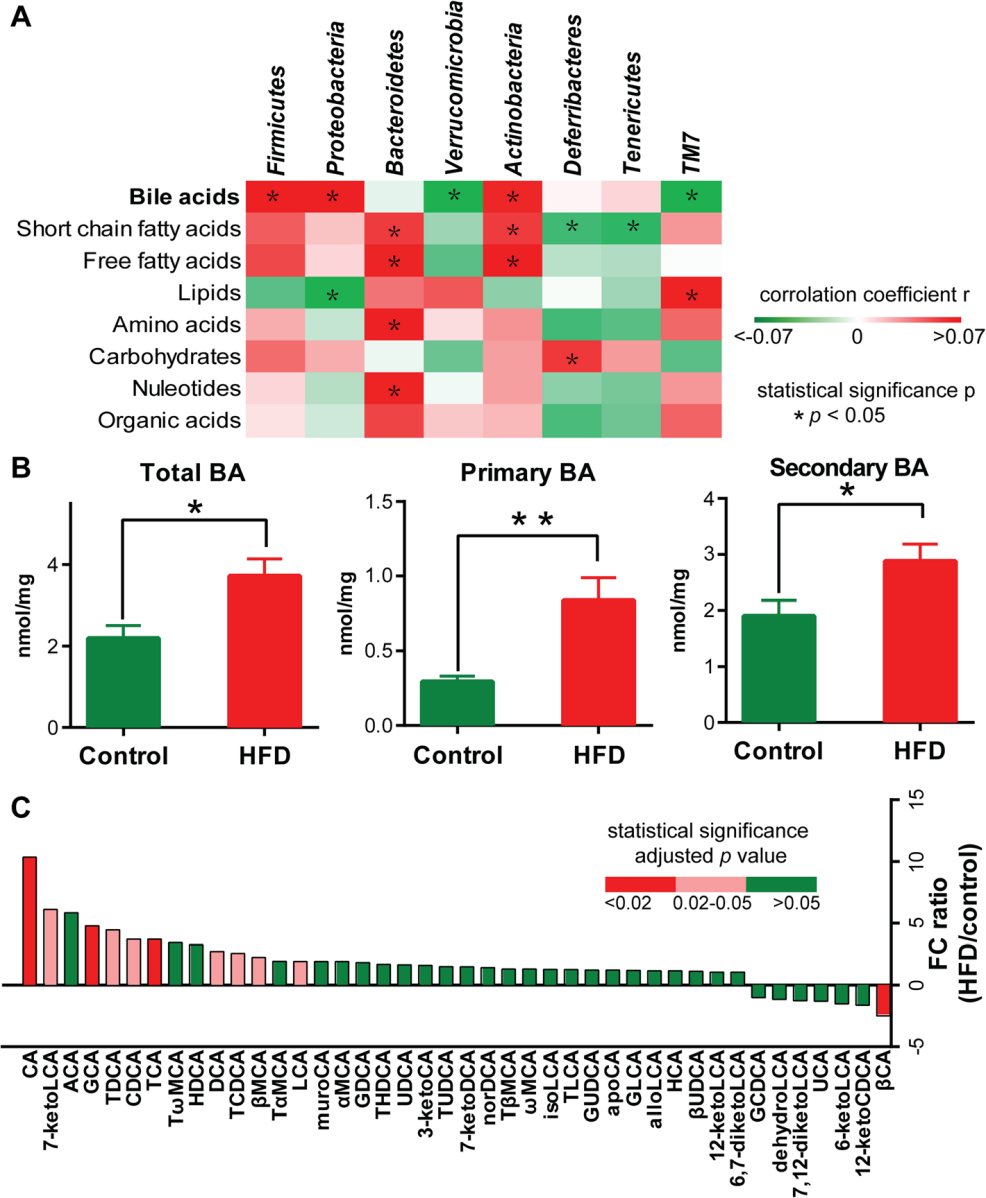
The correlation between metabolite types and gut microbial phyla, and BA alterations with HFD intervention. **a** Principal component analysis was performed on each metabolite type and each phylum of gut microbiome. The *t* values of the first principal component (PC1) among the samples from control (n = 5) and HFD (n = 5) groups were selected as representative of each dataset. The heatmaps depict Spearman correlation of metabolite types and microbial phyla. Red and green cells indicate positive and negative correlations, respectively; *P < 0.05. **b** The concentrations of total, primary, and secondary BAs in caecal content in control (*n* = 7) and HFD (n = 7) groups. Data are expressed as mean ± SEM. Differences were assessed by the Mann–Whitney U test. Significance is established at adjusted *P* < 0.05 (*P < 0.05, **P < 0.01) with a false discovery rate (FDR) of 0.05. **c** The fold change ratios of the average concentrations of identified BA species in the HFD group (n = 7) to that in the control group (n = 7). Differences are assessed by the Mann–Whitney U test. Significance is established at adjusted *P* < 0.05 with FDR = 0.05

Male C57BL/6 mice (3 weeks old) were fed normal chow (control group) and a HFD (HFD group) for 8 weeks prior to sacrifice. The physiological and biochemical parameters of the mice are listed in Additional file 1: Table S1. To establish any correlation of different metabolite types with specific gut microbiota, the global metabolome profiling of caecal content was initially performed using gas chromatography-time-of-flight mass spectrometry (GC/TOFMS), ultra-performance liquid chromatography-triple quadrupole mass spectrometry (UPLC/TQMS), and ultra-performance liquid chromatography-quadrupole time-of-flight mass spectrometry (UPLC/QTOFMS). A total of 211 metabolites were detected, including BAs, short chain fatty acids (SCFAs), free fatty acids, lipids, amino acids, carbohydrates, nucleotides, and organic acids (Additional file 2: Figure S1 and Additional file 3: Table S2). The 16*S* rRNA genes of caecal microbiota were assessed using an Illumina Miseq Platform. To reduce the data dimension for each type of metabolites and gut microbial composition at phylum levels, principal component analysis was performed (Fig. 1a and Additional file 4: Table S3). The results showed that BAs were significantly correlated with five out of eight gut microbe phyla. *Firmicutes*, *Proteobacteria*, and *Actinobacteria* had positive correlations, while *Verrucomicrobia* and *TM7* had negative correlations with BAs. Comparatively, the number of phyla that were significantly correlated with the other metabolite types was lower. These results imply that BAs are the most relevant metabolites affecting gut microbiota. Thus, BAs were identified as significant metabolic factors correlated with the gut microbiota, providing the rationale for further studies of the interaction of BAs with gut microbiota.

To assess the interaction of BAs with caecal microbiota, we quantified the concentration of BAs in the caecal content of mice fed with control chow and HFD, and found that caecal BA levels were dramatically increased in response to HFD (Fig. 1b). Among the 42 quantified BAs, 35 had higher concentrations in the HFD group, including CA, glycocholic acid (GCA), taurodeoxycholic acid (TDCA), TCA, chenodeoxycholic acid (CDCA), deoxycholic acid (DCA), taurochenodeoxycholic acid (TCDCA), β-muricholic acid (βMCA), lithocholic acid (LCA), and 7-ketolithocholic acid (7-ketoLCA), with statistical significance (Fig. 1c and Additional file 5: Table S4). Only one BA, β-cholic acid (βCA), was significantly decreased after a HFD intervention. All of the CA-derived BAs, including CA, TCA, and GCA, were significantly increased, implying that CA-derived BAs might be the important BA species in the response to dietary fat.

### Dietary fat-induced gut microbiome alteration and its correlation with BAs

**Fig. 2:**
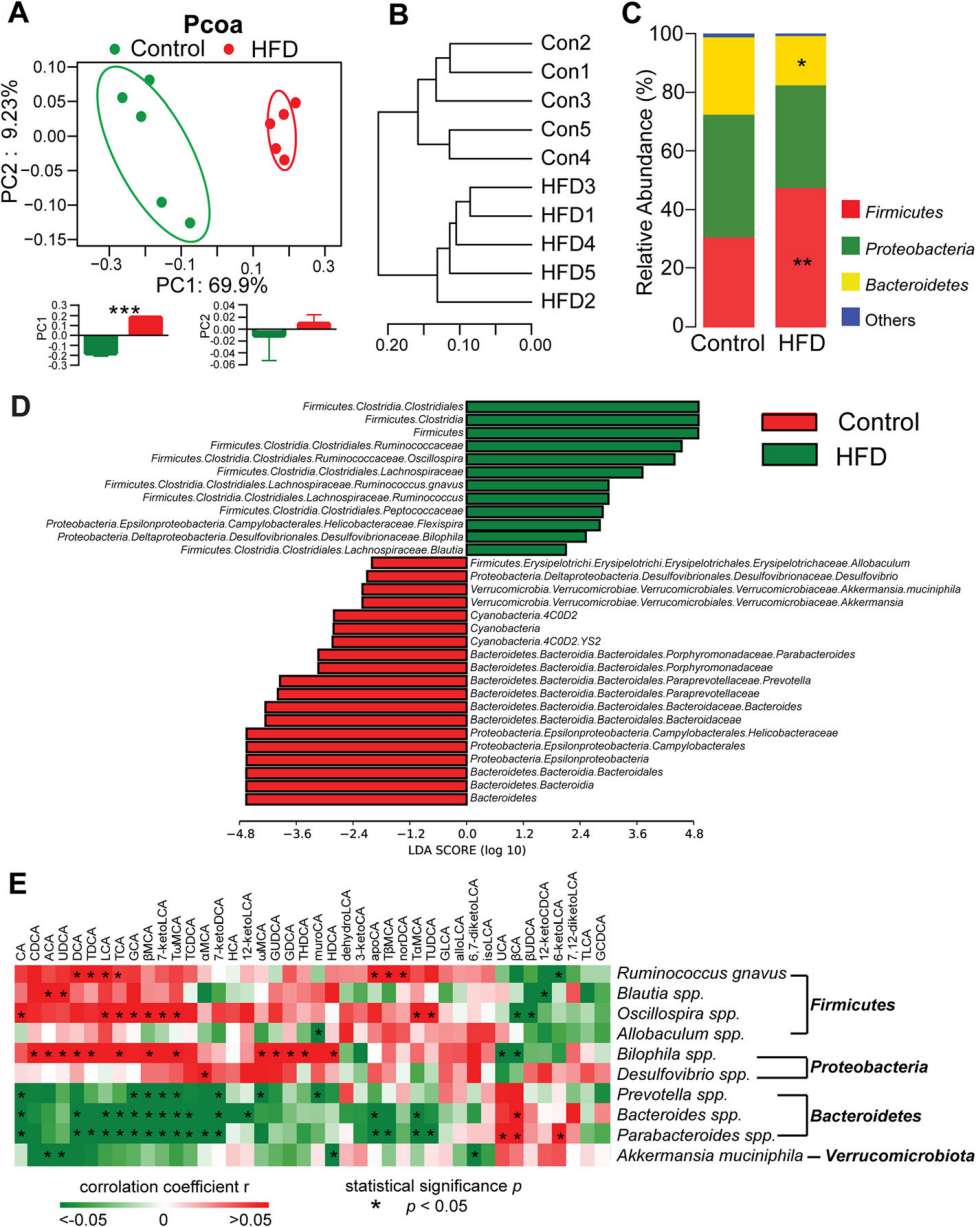
HFD-induced microbial composition changes in mouse caecum. **a** Principle coordinate analysis (PCOA) plot is generated using OTU metrics based on the Bray–Curtis similarity for the samples in control (n = 5) and HFD (n = 5) groups. The center point coordinate of the ellipse is the mean value of PC1 and PC2, respectively, in the corresponding group. The length of the semi-major and semi-minor axes of the ellipse is 1.5-fold SD of PC1 and PC2, respectively. The ellipse is rotated to the direction of largest variation of the corresponding group. The values of PC1 and PC2 are shown in bar plots. Data are expressed as mean ± SEM. Differences are assessed by the Mann–Whitney U test. Significance is established at *P* < 0.05 (***P < 0.001). **b** Hierarchical clustering based on the Bray–Curtis similarity of caecal content microbial composition in control (n = 5) and HFD (n = 5) groups. **c** The mean percentage of the total population at phylum levels in the caecal microbiota in control (n = 5) and HFD (n = 5) groups. Differences are assessed by the Mann–Whitney U test. Significance is established at *P* < 0.05 (*P < 0.05, **P < 0.01). **d** Linear discriminant analysis (LDA) effect size method was performed to compare taxa between control (red; n = 5) and HFD (green; n = 5) groups. The bar plot lists the significantly differential taxa based on effect size (LDA score (log 10) > 2). Enriched taxa in controls (negative LDA score), and enriched taxa in HFD (positive LDA score). **e** Spearman correlations of the relative abundance of differential microbial species selected by the LDA effect size method and the concentrations of BAs among the samples from control (n = 5) and HFD (n = 5) groups. The r values are represented by gradient colors, where red and green cells indicate positive and negative correlations, respectively; *P < 0.05

The impact of a HFD on caecal microbiota composition was revealed by sequencing of the respective 16*S* rRNA genes. The Simpson reciprocal index of diversity in the HFD group was lower than in the control, indicating that the microbial diversity was reduced after a HFD intervention (Additional file 6: Figure S2). An unweighted principle coordinate analysis (PCOA) was conducted to visualize differences in bacterial taxa composition between diet types (Fig. 2a). The PCOA plot indicated an obvious separation between the two groups along the PC1 axis (69.9% of overall variation) with statistical significance. Consistently, the samples in the control and HFD groups exhibited hierarchical clustering based on the Bray–Curtis similarity (Fig. 2b). Population analyses for each diet group were performed and the mean percentage of the total population at phylum levels is shown in Fig. 2c and Additional file 7: Table S5. The results showed that the bacterial population in control caecum was dominated by *Proteobacteria* (41.91%), *Firmicutes* (30.59%), and *Bacteroidetes* (26.22%), together with minority populations such as *Verrucomicrobia* (0.95%), *Deferribacteres* (0.12%), and *Cyanobacteria* (0.018%). Conversely, in the HFD-fed group, *Firmicutes* expanded significantly (47.51%) at the expense of *Proteobacteria* (34.86%) and *Bacteroidetes* (16.94%). To elucidate the main altered microbes after the HFD intervention, we used the linear discriminant analysis effect size method to compare taxa between the control and HFD groups (Fig. 2d, Additional file 8: Figure S3, and Additional file 9: Table S6). This analysis revealed that the increase in *Firmicutes* resulted from expansion of class *Clostridia* and order *Clostridiales*. The decrease in *Bacteroidetes* resulted from a reduction of class *Bacteroidia* and order *Bacteroidales*. The decrease in *Proteobacteria* resulted from a reduction of class *Epsiloproteobacteria* and order *Campylobacterales*. Ten microbes at species and genus levels were significantly altered among these altered taxa. Further, *Ruminococcus gnavus*, *Blautia spp*., and *Oscillospira spp*. under the phylum *Firmicutes*, and *Bilophila spp.* under the phylum *Proteobacteria* were significantly higher in the HFD group. Conversely, *Allobaculum spp*. under the phylum *Firmicutes*, *Desulfovibrio spp.* under the phylum *Proteobacteria*, *Prevotella spp.*, *Bacteroides spp*., and *Parabacteroides spp*. under the phylum *Bacteroidetes*, and *Akkermansia muciniphila* under the phylum *Verrucomicrobia* were significantly lower.

To visualize the correlation of BAs and gut microbiota, a Spearman correlation was performed between the relative abundance of the 10 differential microbial species and the concentrations of all BAs across the samples in the control and HFD groups (Fig. 2e and Additional file 10: Table S7). Among the 42 BAs shown in the heatmap, 33 had at least one significant correlation with a microbe. Most microbes in *Bacteroidetes* and *Verrucomicrobia* phyla were negatively correlated with BA changes, while those in *Firumicutes* and *Proteobacteria* phyla were positively correlated with BA changes.

### How BAs alter the gut microbial composition

In order to determine the temporal relationship between BA exposure and alterations in intestinal microbial composition in response to HFD, we performed time-dependent studies on BAs and gut microbiome composition with HFD intervention. Mice fed with HFD or normal chow were sacrificed at different time points, i.e., at days 0, 0.5, 1, 2, 3, 6, 10, 28, and 56.

**Fig. 3:**
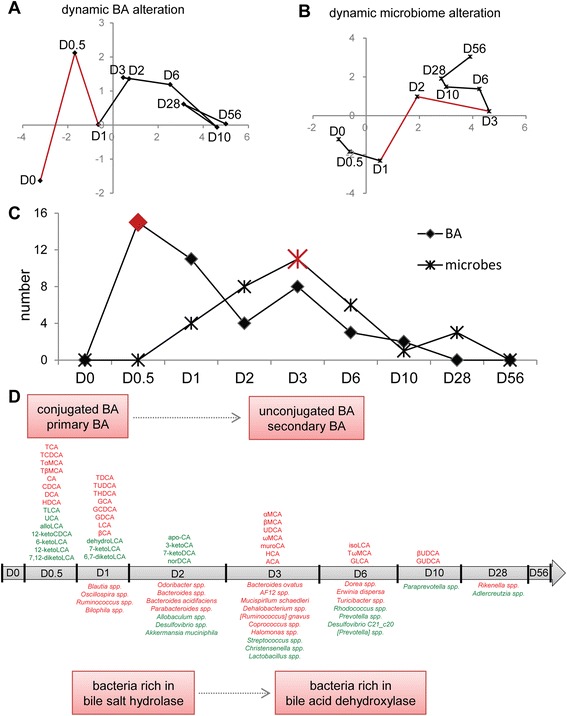
The time-dependent alteration of BAs and microbe species in response to HFD. **a** Alteration of BAs shown by trajectories based on *t* values of principal component 1 (PC1) and PC2 of partial least squares discriminant analysis (PLS-DA). The red lines from D0 to D0.5 and D0.5 to D1 highlight when the most dramatic changes occurred. **b** Alteration of microbiota shown by trajectories based on *t* values of PC1 and PC2 of PLS-DA. The red lines from D1 to D2 and D2 to D3 highlight when the most dramatic changes occurred. **c** The concentration of each BA and the abundance of microbe species were compared between control (n = 5) and HFD (n = 5) groups at the corresponding time points. The BAs and microbes were recorded when they were significantly altered at the earliest time point. The number of recorded BAs and microbes is shown in the line charts, and the peaks of the two lines are highlighted in red. **d** The BAs and microbes selected in Fig. 4c are listed along the timeline, where red and green indicate high and low concentrations, respectively, in the HFD group relative to the control group at each corresponding time point

To observe the overall alterations of BA and gut microbiota at various time-points, multivariate statistics partial least squares discriminant analysis was performed. The results showed that BAs were significantly altered during the first day of HFD intervention (Fig. 3a and Additional file 11: Figure S4), while microbiota fluctuated dramatically from D1 to D3 (Fig. 3b and Additional file 11: Figure S4).

The concentrations of BAs and the abundance of gut microbes in both the control and HFD groups were compared at each corresponding time point. The concentrations of BAs, as well as all of the microbes at species and genus levels, were recorded when they were significantly altered at the earliest time point. The number of BAs reached a plateau after only 12 h of HFD intervention, indicating that a HFD rapidly stimulates BA secretion (Fig. 3c). However, there were no changes in the microbiota detected during this period. The number of microbes gradually increased and reached their highest value at D3. Such observations suggest that the response to BAs precedes and perhaps affects the gut microbiota response to the HFD intervention. Regarding the specific BAs (Fig. 3d and Additional file 12: Table S8), after 1 day of the HFD intervention, the concentration of 26 BAs were altered, and the concentrations of conjugated BAs TCA, TCDCA, tauro-α-muricholic acid (TαMCA), tauro-β-muricholic acid (TβMCA), GCA, and glycochenodeoxycholic acid (GCDCA) were increased. The unconjugated BAs could be sorted into two distinct groups conformed of most primary BAs, which had an increased concentration, and most secondary BAs, which had a decreased concentration. The concentrations of specific microbes, such as *Bilophila spp.*, *Blautia spp.*, *Oscillospira spp*., and *Ruminococcus spp*., were initially increased. The alteration of the microbial community in the caecum was then reshaped, with some species coming more prominent and others less so. After 10 days of the HFD intervention, all of the detected BAs had fluctuated, while two microbes were observed to be impacted at D28. The response of gut microbiota alteration lagged behind that of BA increase, implying that BAs might regulate the composition of gut microbiota.

### BAs alone induce the obesity-associated gut microbial composition

To confirm that the HFD-induced gut microbial alterations were correlated with BAs, BAs were administered to mice under normal diet conditions. GCA and TCA were selected as the BA supplementation for two reasons. Firstly, CA-type BAs were significantly increased after the HFD intervention, implying the predominant role of these BAs is associated with dietary fat. Secondly, conjugated primary BAs were the main components of bile secreted to intestine in response to dietary fat.

**Fig. 4:**
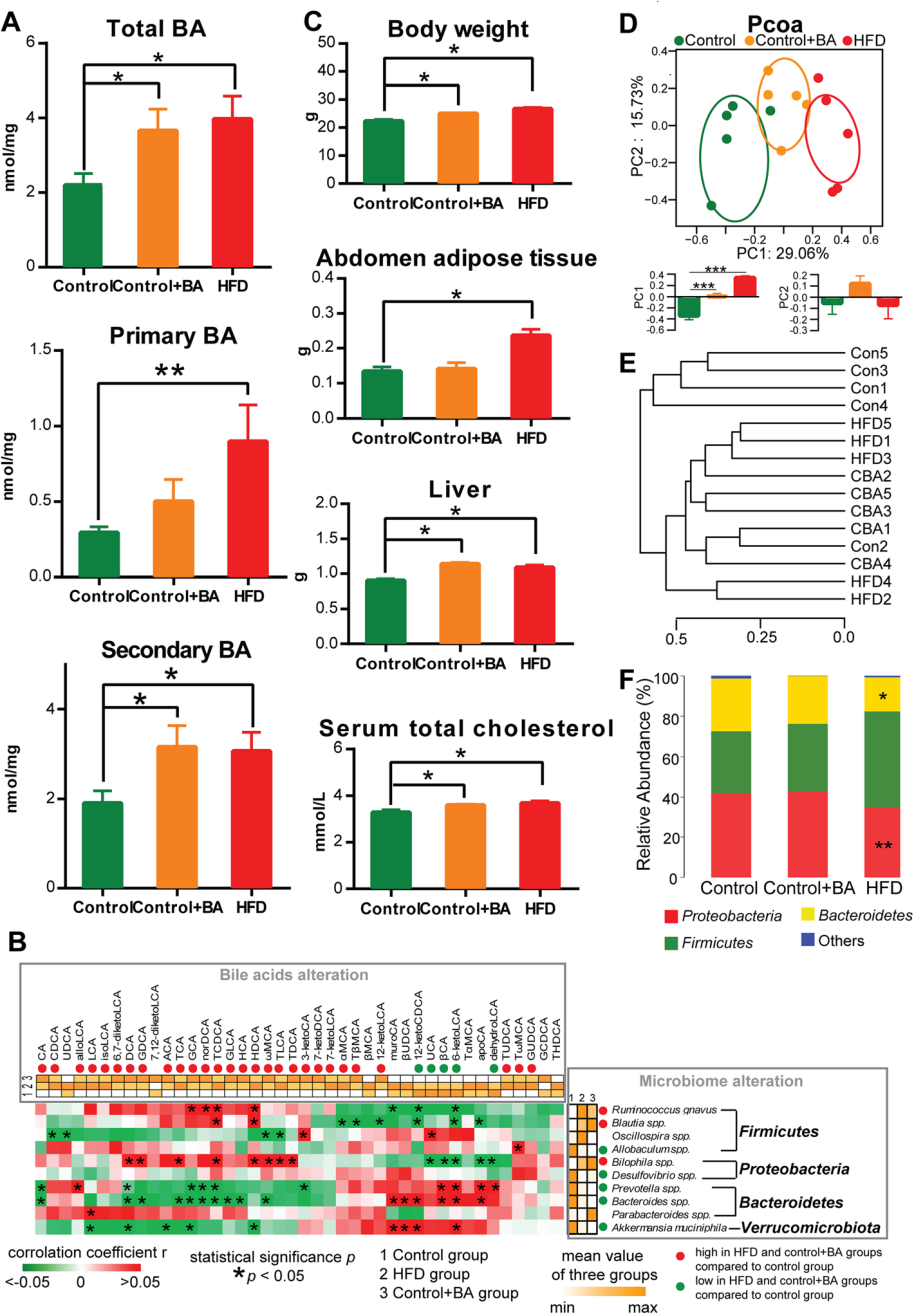
The concentrations of BAs, physiological changes, and microbial composition after HFD feeding or BA supplementation. **a** The concentrations of total BAs, primary BAs, and secondary BAs in caecal content in control, control + BA, and HFD groups (n = 7 per group). Data are expressed as mean ± SEM. Differences were assessed by the Mann–Whitney U test. Significance was established at adjusted *P* < 0.05 (*P < 0.05, **P < 0.01) with FDR = 0.05. **b** Heatmaps of BA alteration, microbiome alteration, and their correlations. The two heatmaps with gradient colors of white to orange illustrate the mean concentration of BAs and the mean abundance of microbes in control, HFD, and control + BA groups. The heatmaps with gradient colors of green, white, and red illustrate the Spearman correlations of the relative abundance of microbes and the concentrations of BAs among the samples from the control (n = 5) and control + BA (n = 5) groups. The r values are represented by gradient colors, where red and green cells indicate positive and negative correlations, respectively; *P < 0.05. **c** Body weight, abdomen adipose tissue weight, liver weight, and the concentration of serum total cholesterol in control, control + BA, and HFD groups (n = 7 per group). Differences are assessed by the Mann–Whitney U test. Significance is established at adjusted *P* < 0.05 (*P < 0.05) with FDR = 0.05. **d** Principle coordinate analysis (PCOA) plot is generated using OTU metrics based on the Bray–Curtis similarity for the samples in control, control + BA, and HFD groups (n = 5 per group). The center point coordinate of the ellipse is the mean value of PC1 and PC2, respectively, in the corresponding group. The length of the semi-major axis and semi-minor axes of the ellipse is 1.5-fold SD of PC1 and PC2, respectively. The ellipse is rotated to the direction of largest variation of the corresponding group. The values of PC1 and PC2 are shown in bar plots. Data are expressed as mean ± SEM. Differences were assessed by the Mann–Whitney U test. Significance was established at *P* < 0.05 (***P < 0.001). **e** Hierarchical clustering based on the Bray–Curtis similarity for the microbial composition of caecal content of each sample in control, control + BA, and HFD groups (n = 5 per group). **f** The mean percentage of the total population at phylum levels in the caecal microbiota in control, control + BA, and HFD groups (n = 5 per group). Differences were assessed by the Mann–Whitney U test. Significance was established at *P* < 0.05 (*P < 0.05, **P < 0.01)

Feeding mice with GCA and TCA changed the host BA profiles dramatically. The total BA pool was increased, especially for secondary BAs (Fig. 4a). Most BA species were markedly increased following GCA and TCA supplementation (Additional file 5: Table S4), similarly to what was observed in the HFD study, as evidenced by the BA concentration heatmap (Fig. 4b) in ‘Bile acids alteration’, showing that 33 BAs among the detected 42 had the same alteration trend in the HFD and control + BA groups compared to the control group.

We also observed that supplementation of BAs significantly changed the phenotype and biochemical parameters of mice (Fig. 4c and Additional file 1: Table S1), similarly to observations in the HFD study, i.e., increased body weight gain, fat mass, liver weight, and serum total cholesterol. The results confirmed the correlative relationship between phenotype alteration and BA administration.

The impact of BAs on the caecal microbiota composition was revealed by sequencing of 16*S* rRNA genes. The Simpson reciprocal index for gut microbiota alpha diversity was decreased in the control + BA group, even lower than that determined for the HFD group relative to the control group (Additional file 13: Figure S5). Microbial composition differences could be visualized in the PCOA plot (Fig. 4d), which reveals clear separation among all three groups. The distribution of the samples in the control + BA group were between those in the control and HFD groups, and had statistical significance along the PC1 axis compared to the control group. This indicated that the microbiota composition with normal chow had been altered with BA impact in a similar way as observed with a HFD. Consistently, the samples in the control + BA and HFD groups were clustered to the same node, separately from the control, except for one control sample (Fig. 4e). Thus, BA administration can directly alter gut microbial composition, mimicking the HFD intervention. Population analyses for each diet group were performed and the mean percentage of the total population at phylum levels is shown in Fig. 4f and Additional file 7: Table S5. The results showed that, relative to the control, the gastrointestinal microbiota in the control + BA group was characterized by a reduction in *Bacteroidetes* (26.22% to 23.53%) and an increase in *Firmicutes* (30.59% to 33.24%) although without statistical significance. *Verrucomicrobia*, which has relatively low abundance in the gut microbial composition, was significantly decreased in the control + BA group compared to the control. This decrease was similar to that observed for a HFD. The *Proteobacteria* phylum was increased after BA intervention but not after HFD treatment.

To visualize the impact of BAs on the 10 microbial species significantly changed by HFD, we compared the mean value of abundance of each microbe in the control, HFD, and control + BA groups. The results (Fig. 4b) in ‘Microbiome alteration’ showed that eight of the 10 microbes had the same alteration trend in the HFD and control + BA groups compared to the control. The alterations of *Oscillospira spp.* and *Parabacteroides spp.* were inconsistent, implying that the alterations of these two microbes by HFD might be due to other causes. The resulting microbial composition implied that the eight microbial species *R. gnavus*, *Blautia spp.*, *Allobaculum spp.*, *Bilophila spp.*, *Desulfovibrio spp.*, *Prevotella spp.*, *Bacteroides spp.*, and *Akkermansia muciniphila*, might be regulated by BAs in the obesity-associated gut microbiome.

We then performed a Spearman correlation between the BAs and the microbes in the control and control + BA groups. We observed that, except for *Desulfovibrio spp*., the rest of the seven microbes were significantly correlated with specific BAs (Fig. 4b and Additional file 14: Table S9). Thus, these seven microbes might be the bacteria that differentially respond to increases of a specific HFD-induced BA secretion.

### BA reduction as a therapeutic option for attenuating HFD-induced gut microbiome alteration and obese phenotype

The HFD effects on obesity might be due to its effects on BA pool size. Therefore, we inhibited hepatic BA biosynthesis by using the synthetic farnesoid X receptor (FXR) agonist, GW4064 [11], in male C57BL/6 J mice. Meanwhile, to confirm that the impact of HFD-induced BA secretion on the obesity phenotype was regulated by gut microbiota, the broad-spectrum and non-absorbable antibiotic rifaximin was administered to mice under HFD conditions.

**Fig. 5:**
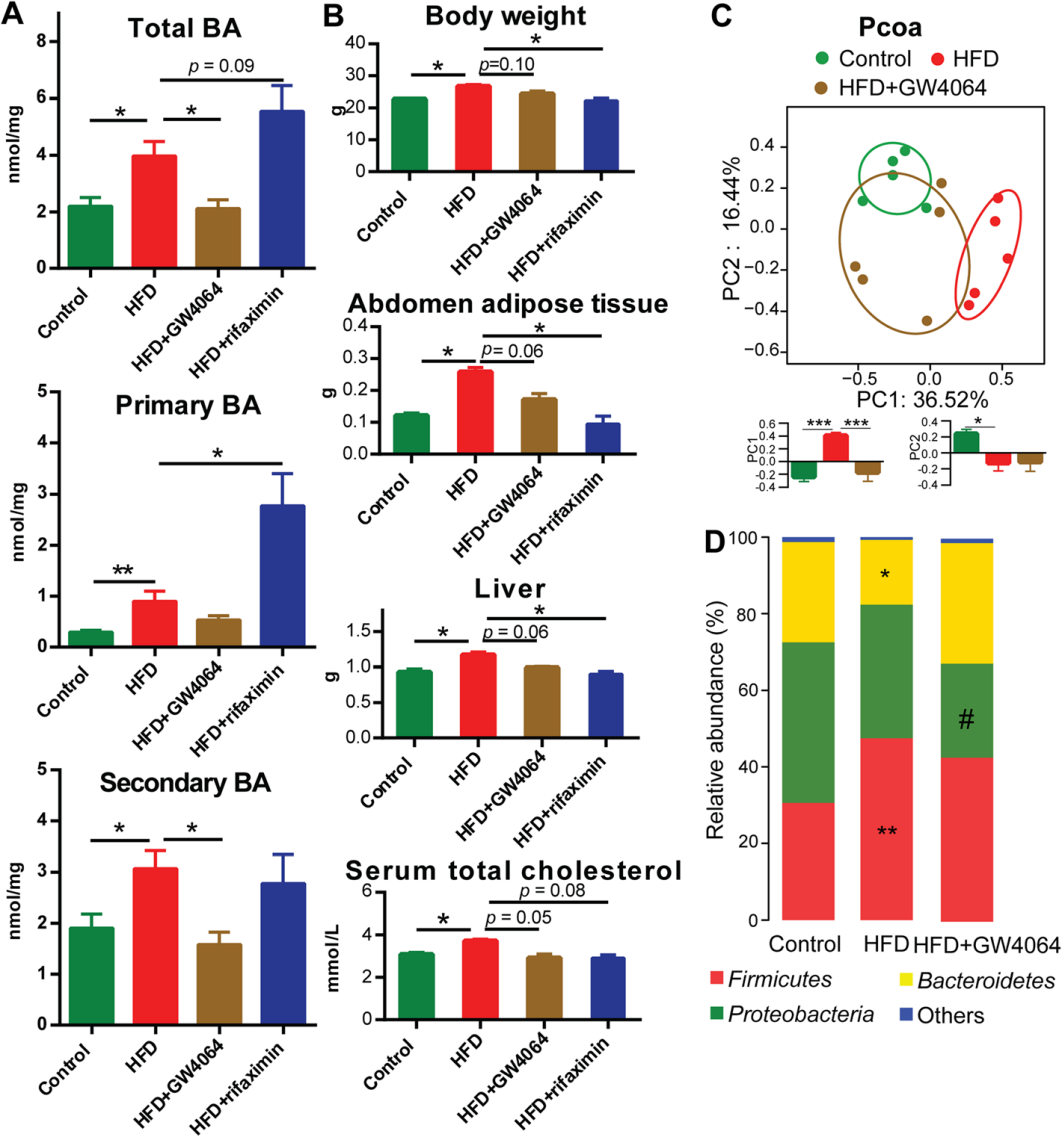
The concentrations of BAs, physiological changes, and microbial composition after HFD, HFD + GW4064, and HFD + rifaximin feeding. **a** The concentrations of total BAs, primary, and secondary BAs in caecal content in control, HFD, HFD + GW4064, and HFD + rifaximin groups (n = 7 per group). Data are expressed as mean ± SEM. Differences were assessed by the Mann–Whitney U test. Significance was established at adjusted *P* < 0.05 (*P < 0.05, **P < 0.01) with a false discovery rate (FDR) of 0.05. **b** Body weight, abdomen adipose tissue weight, liver weight, and concentration of total serum cholesterol in control, HFD, HFD + GW4064, and HFD + rifaximin groups (n = 7 per group). Data are expressed as mean ± SEM. Differences were assessed by Mann–Whitney U test. Significance was established at adjusted *P* < 0.05 (*P < 0.05) with FDR = 0.05. **c** Principle coordinate analysis (PCOA) plot generated using OTU metrics based on the Bray–Curtis similarity for control, HFD, and HFD + GW4064 groups (n = 5 per group). The center point coordinate of the ellipse is the mean value of PC1 and PC2, respectively, in the corresponding group. The length of the semi-major and semi-minor axes of the ellipse is 1.5-fold SD of PC1 and PC2, respectively. The ellipse is rotated to the direction of largest variation of the corresponding group. The values of PC1 and PC2 are shown in bar plots. Data are expressed as mean ± SEM. Differences were assessed by the Mann–Whitney U test. Significance was established at *P* < 0.05 (*P < 0.05, ***P < 0.001). **d** Mean percentage of the total population at phylum levels in the caecal microbiota in control, HFD, and HFD + GW4064 groups (n = 5 per group). Differences were assessed by the Mann–Whitney U test. Significance was established at *P* < 0.05 (control vs. HFD: *P < 0.05, ** *P* < 0.01; HFD vs. HFD + GW4064: #*P* < 0.05)

As expected, GW4064 administration attenuated the BA increase in mice after HFD feeding, especially the levels of total and secondary BAs. With suppression of gut microbiota by antibiotic, the BAs, especially primary BAs, were significantly increased compared to those in the HFD group (Fig. 5a and Additional file 5: Table S4).

The mice fed with a HFD supplemented with GW4064 did not gain weight as rapidly as HFD controls based on their similar energy intake. Such intervention also induced a lower fat mass and liver weight. In addition, GW4064 supplementation counteracted the HFD-induced high serum total cholesterol. Similarly, we also observed a reduction in body weight, abdomen adipose tissue weight, liver weight, and serum total cholesterol concentration in the HFD + rifaximin group (Fig. 5b and Additional file 1: Table S1).

Gut microbiota fluctuated after GW4064 administration relative to the HFD group. The alpha diversity was significantly increased after GW4064-induced BA regulation (Additional file 15: Figure S6). A preliminary unweighted PCOA (Fig. 5c) was conducted to visualize differences in bacterial taxa composition and the trend indicated that the HFD + GW4064 group was closer to the controls than to those in the HFD group with statistical significance along the PC1 axis between the HFD and HFD + GW4064 groups. This implied that the composition of obesity-associated gut microbiota has been reversed towards the control state after BA regulation. The phylum bar plot also revealed that the composition of gut microbiota in HFD + GW4064 was similar to controls. The abundance of *Firmicutes* and *Proteobacteria* were decreased, while *Bacteroidetes* and *Verrucomicrobia* were increased in the HFD + GW4064 compared to HFD groups (Fig. 5d and Additional file 7: Table S5). Thus, utilization of an FXR agonist may prevent or reverse HFD-induced gut microbiome alterations and impair the development of obese phenotypes.

## Discussion

Diet can affect the intestinal microbiota composition and activity in various ways, including providing prebiotic substrates and other fermentable constituents, regulating transit time, pH, and host secretions, as well as influencing gene expression in both the host and microbiome [12]. Based on the global metabolome analysis and multivariate statistics performed herein, BAs showed a predominant role in host–microbiota co-metabolism, confirming that BAs are a significant dietary factor influencing the composition of gut microbiota.

Previous reports have shown that a HFD changes the relative composition of gut microbiota by increasing *Firmicutes* and decreasing *Bacteroidetes* at phylum levels along with alterations of the microbes at species levels such as *Bilophila* and *Bacteroides* [1, 2, 7, 13]. Our results confirmed a similar shift in population between *Bilophila spp*., *Firmicutes*, and *Bacteroidetes*. The interaction of BAs and the gut microbiome is well-known [8]; however, herein, the link between exposure to specific types of BAs and specific changes in gut microbiota was revealed in a time course experiment that clearly demonstrated that the HFD-induced secretion of BAs into the intestine maximized at 12 h post-HFD and preceded the changes in microbiota, which maximized at 72 h. These results thus demonstrate that BAs are responsible for microbiota composition changes. Further confirmation for this effect of BAs came from our follow-up experiment, which reproduced the HFD phenotype via replacement of a HFD with BA feeding. To our knowledge, this type of time-dependent experiment correlating changes in BAs with those of microbiota has not been previously published.

Seven microbial species were found to have similar responses to both HFD and BA administration. Among these seven microbial species, three were increased with the HFD intervention, including *Bilophila spp.*, *Ruminococcus spp.*, and *Blautia spp.* increasing after 1 day of intervention and, specifically, *R. gnavus* increasing at day 3. Such alterations suggested that these three types of microbes were bile tolerant. On the other hand, the decrease in populations of *Prevotella*, *Bacteroides*, and *Parabacteroides spp.* as well as *A. muciniphila* implied that these bacteria were more sensitive to BAs.

Previous research [7] has shown that *Bilophila wadsworthia* was stimulated in mice by BA secretion. This microbe is a sulfite-reducing bacterium that thrives in the presence of TCA. A previous clinical study [1] also supported our results that *Bilophila spp.* growth in humans could be promoted by a HFD. The byproducts of *Bilophila spp.*, H_2_S and secondary BAs, have been shown to perturb the immune homeostasis and induce colonic tissue damage, suggesting that the increase in *Bilophila spp.* might be involved in the pathogenesis of colonic diseases in obese individuals [7]. *Blautia spp.* were found to be capable of 7α-dehydroxylation to produce the more anti-microbial secondary BAs from primary BAs [14]. Secondary BAs are known to be high affinity ligands for FXR [15]. BA activation of FXR downregulates BA synthesis and stimulates the synthesis of BA-detoxifying enzymes. Activated FXR can also bind directly to the pro-inflammatory transcription NF-κB and control intestinal inflammation. The increase in *Blautia spp.* after the first day may therefore play an important role as a compensating response to the presence of increased amounts of BAs in the gut [15–18]. *R. gnavus* has been reported to be an ursodeoxycholic acid (UDCA) producer as it produces 7β-hydroxysteroid dehydrogenase, an enzyme capable of degrading 7-ketoLCA to UDCA [19]. Increased *R. gnavus* along with decreased levels of 7-ketoLCA and increased UDCA levels at day 1 were observed herein. UDCA is cytoprotective against secondary BAs such as DCA and has been previously shown to be able to inhibit DCA-induced activation of the EGFR/Raf-1/ERK signaling pathway in HCT-116 cells and in an azoxymethane mouse model for colorectal cancer [20, 21]. *R. gnavus* is also involved in the iso-BA pathway, which detoxifies secondary BAs producing iso-BAs with lower detergent and cytotoxic activities [22]. Our data showed a positive correlation trend between increased *R. gnavus* and isolithocholic acid levels.

Four microbes were decreased in our study, *A. muciniphila*, *Allobaculum spp*., *Desulfovibrio spp.*, and *Prevotella spp.*, all of which are considered beneficial bacteria commonly higher in healthy relative to obese people [23–25]. *A. muciniphila*, a mucin-degrading bacterium, has been reported to exert an anti-obesogenic effect and has been shown to be significantly decreased in obese animals [23, 26]. Administration of *A. muciniphila* reversed HFD-induced fat-mass gain, metabolic endotoxemia, adipose tissue inflammation, and insulin resistance [24]. *Desulfovibrio spp.* are also involved in mucin fermentation and found to be more abundant in normal weight children compared to those who are overweight [25]. *Allobaculum* and *Prevotella spp*. [27] degrade dietary fiber to SCFAs, which serves as a main colonic energy source. *Prevotella spp*. help maximize energy extraction from a diet rich in fiber [28]. The abundance of *Allobaculum spp*. was previously shown to be strongly inversely correlated with the amounts of circulating leptin and the expression of several genes correlated with energy expenditure, homeostasis, and inflammation.

Overall, our findings showed that the presence of increased populations of bacteria that are capable of deconjugating and producing secondary BAs in response to high concentrations of BAs may provide some protective compensation effects via increased potential for FXR activation and UDCA production. Simultaneously, there were decreased populations of beneficial microorganisms that are anti-obesogenic in response to increased BA exposure.

A previous study modified obese mouse phenotypes using probiotics [26], and provided evidence that stimulation of beneficial bacterial growth may occur along with depression of more harmful bacteria by lowering BA secretion through inhibition of BA biosynthesis. In the present study, the use of the FXR agonist GW4064 to inhibit BA biosynthesis prevented the development of the obese phenotype and its corresponding alteration of the gut microbial composition. Further, the HFD led to increased BA secretion into the gut, whereas the normal diet did not, thus a dietary intervention may also be effective in altering the obese phenotype.

The alteration trends of *Bacteroides spp.* varied between the short- and long-term HFD interventions. *Bacteroides spp.* concentrations were increased at day 2 but were low at 8 weeks. Previous reports revealed *Bacteoriodes spp*. as bile-tolerant microbes [1], becoming increased during the first several days following exposure to high levels of BAs. Our results indicated that BAs could have a varying selective pressure on the different bile tolerant microbes. Some microbes were resistant to apoptosis, and were even highly favored in the presence of high BA concentrations, whereas other microbes such as *Actinobacter spp*. showed an increased abundance only at low BA concentrations (day 28). With the high concentration of conjugated and primary BAs, those bacteria containing the enzymatic machinery for bile salt dehydrolase and dehydroxylation showed increased growth. The observed increase in hydrophobic secondary BAs, which are strong antimicrobial compounds, is a reasonable explanation for the suppression of beneficial bacteria that were bile intolerant.

The fact that all of the animal models used were male mice represents a limitation to the present study. Considering the sex-based differences in BA patterns and gut microbial composition, future studies on both sexes are required.

## Conclusions

Our study provided a correlative relationship between BAs and the HFD-induced alteration of gut microbiota. Dietary intervention and FXR activation has been confirmed to play an important role in the treatment of obesity via a decrease in BA secretion and/or synthesis. Future studies will be needed to establish similar relationships between changes in both BA concentrations and composition and changes in the gut microbiota in human obese versus normal weight phenotypes. Ultimately, identification of key microorganisms may yield better probiotic therapeutics to replenish beneficial bacteria that have been lost by patients suffering from obesity and metabolic syndrome. The development of safe FXR agonists along with dietary consultation will translate our experimental findings into clinical practice.

## Methods

### Animal studies and sample collection

All animal studies were performed following national legislation and local guidelines at the Center for Laboratory Animals, Shanghai Jiao Tong University, Shanghai, China. C57BL/6 J mice (male, 3 weeks old) were purchased from Shanghai Laboratory Animal Co. Ltd. (SLAC, Shanghai, China) with 1 week of acclimatization. All experimental mice were housed in specific pathogen-free environments under a controlled condition of 12 h light/12 h dark cycle at 20–22 °C and 45 ± 5% humidity, with free access to chow and ultrapure water. The mice were randomly divided into groups for further experiments. The body weights and food intake of all animals were recorded once a week during the experiments. Mice were fasted overnight and then anesthetized for blood harvesting. The blood was centrifuged at 3,000 *g* for 15 min at 4 °C for serum collection. Livers, three types of white adipose tissues (epididymal, abdomen, and kidney adipose tissues), brown adipose tissues, and caecal content were carefully dissected and kept in liquid nitrogen before storage at −80 °C.

#### Animal experiment 1

Mice were divided into five groups (n = 7 per group as biological replicates) with different diets for 8 weeks: (1) control group, fed with normal chow (10% fat calories); (2) HFD group, fed with diet containing 45% fat calories from coconut oil; (3) control + BA group, fed with control chow supplemented with 1 mmol/kg GCA and TCA, respectively; (4) HFD + GW4064 group, fed with HFD supplemented with 180 mg/kg GW4064; and (5) HFD + rafiximin group, fed with HFD supplemented with 80 mg/kg antibiotic rafiximin. The feed ingredients of normal chow and HFD are provided in Additional file 16: Table S10.

#### Animal experiment 2

Mice were divided into 18 groups (n = 5 per group as biological replicates), of which nine were fed with normal chow and nine were fed a HFD. Two groups of mice, one each for the different diets, were sacrificed at each of the following nine time points: days 0, 0.5, 1, 2, 3, 6, 10, 28, and 56.

### Biochemical analysis

The levels of lipopolysaccharides in serum, livers, and caecal content were determined using a mouse lipopolysaccharide Elisa kit (BlueGene Biotech, Shanghai, China) according to the manufacturer’s protocol. Caecal content pH was determined using a pH meter (Mettler-Toledo, Beaumont Leys, UK). Fasting blood glucose was measured using blood glucose monitors (Johnson & Johnson, USA). The analysis of serum triglyceride, total cholesterol, low-density lipoprotein cholesterol, high-density lipoprotein cholesterol, aspartate aminotransferase, alanine aminotransferase, alkaline phosphatase, cholinesterase, total bilirubin, and direct bilirubin was performed using an automatic biochemical analyzer (Hitachi 7600, Tokyo, Japan) [29, 30].

### BA, SCFA, free fatty acid, and metabolite profile assessment

The metabolite assessment in caecal content was based on the protocols established by our lab [31–35]. All of the samples were run in a randomized order to minimize the systematic analytical error. Three samples were selected randomly from each group and were pooled as quality control samples for metabolite profile assessment. The samples were run in triplicate as analytical replicates.

#### BA assessment

Each 10-mg of caecal content sample was extracted by a two-step extraction. A 200-μL aliquot of methanol/water (1:1, containing the six internal standards CA-d4, LCA-d4, UDCA-d4, GCA-d4, GCDCA-d4, and glycodeoxycholic acid-d4, at 50 nM each) was added to the sample. The sample was homogenized for 5 min and centrifuged at 13,200 *g* at 4 °C for 15 min. The supernatant was transferred into a 1.5-mL tube and the sample residue was further extracted by a 200-μL aliquot of methanol/acetonitrile (2:8, containing the six internal standards as the first extraction solvent). After homogenization and centrifugation, the supernatant was transferred to the tube with the first extraction. The extraction mixture was vortexed for 3 min and centrifuged at 13,200 *g* at 4 °C for 15 min. The supernatant was then transferred to a sampling vial for analysis.

BA analysis was performed on the instrument UPLC/TQMS. The elution solvents were water + 0.01% formic acid (A) and acetonitrile/methanol (19:1) + 0.01% formic acid (B). The elution gradient over 20 min at a flow rate of 450 μL/min was as follows: 0–2 min (20% B), 2–3 min (20–25% B), 3–6 min (25% B), 6–8 min (25–35% B), 8–11.5 min (35% B), 11.5–18 min (35–99% B), 18–19 min (99% B), and 19–20 min (99–20% B). The MS was operated at a negative electrospray ionization mode. The cone and collision energy for each BA used the optimized settings from QuanOptimize application manager (Waters Corp., Milford, MA, USA). One standard calibration solution at 10 different concentration levels contains 45 standards and was tested every 40 samples. The peak annotation and quantitation was performed by TargetLynx application manager (Waters Corp.).

#### SCFA assessment

A 500-μL aliquot of 0.005 M aqueous NaOH containing one internal standard (5 μg/mL pentanoic acid-d3) was added to 10 mg of caecal content sample, homogenized for 5 min, and centrifuged at 13,200 *g* at 4 °C for 15 min. The supernatant was transferred into a 10-mL glass centrifuge tube. A 500-μL aliquot of propanol/pyridine mixture solvent (3:2) and 100 μL of propyl chloroformate were subsequently added to the glass tube. After brief vortexing, the derivatization reaction proceeded under ultrasonication for 1 min. After derivatization, the derivatives were extracted by a two-step extraction with hexane. An aliquot of 300-μL hexane was added to the reaction mixture and the sample was vortexed for 1 min followed by centrifugation at 2,000 *g* for 5 min. A 300-μL aliquot of derivative extraction (upper hexane layer) was transferred to a sampling vial. The extraction procedure was then repeated by adding 200 μL instead of 300 μL of hexane to the reaction mixture. Another 200-μL aliquot of derivative extraction was transferred to the sampling vial with the first extraction. Anhydrous sodium sulfate (~10 mg) was added to remove traces of water from hexane. The resultant mixture was briefly vortexed prior to analysis.

SCFAs analysis was performed using GC-TOFMS. Derivatives were separated using an HP-5 ms capillary column coated with 5% phenyl/95% methylpolysiloxane (30 m × 250 μm i.d., 0.25 μm film thickness, Agilent J & W Scientific, Folsom, CA, USA). One microliter of derivatives was injected in split mode with a ratio of 10:1, and the solvent delay time was set to 2.2 min. The initial oven temperature was held at 50 °C for 2 min, ramped to 70 °C at a rate of 10 °C/min, to 85 °C at a rate of 3 °C/min, to 110 °C at a rate of 5 °C/min, to 290 °C at a rate of 30 °C/min, and finally held at 290 °C for 8 min. Helium was used as a carrier gas at a constant flow rate of 1 mL/min through the column. The temperatures of the front inlet, transfer line, and electron impact ion source were set at 260, 290, and 230 °C, respectively. The electron energy was –70 eV, and the mass spectral data was collected in a full scan mode (m/z 30–600). One standard calibration solution at seven different concentration levels contains three standards and was tested every five samples.

The raw data were subject to processing, including baseline correction, smoothing, noise reduction, deconvolution, library searching, and area calculation. Compound identification was performed by comparing both MS spectra and retention times with those of standard compounds. The peak area of each derivatized SCFA was calculated using the unique mass selected by ChromaTOF.

#### Free fatty acids assessment

A 500-μL aliquot of isopropynal/hexane (4:1) with 2% phosphate (2 M) and 10 μL of one internal standard (5 μg/mL of nonadecylic acid-d37) was added to 10 mg of caecal content sample, homogenized for 5 min, and centrifuged at 13,200 *g* at 4 °C for 15 min. A total of 400 μL supernatant was transferred into a 1.5-mL tube. An aliquot of 800-μL hexane and 300 μL of water were then added to the tube, and the mixture was vortexed for 2 min and centrifuged for 10 min at 12,000 *g*. An aliquot of 800 μL upper layer was transferred to a new tube and dried under vacuum. The residue was reconstituted with 80 μL of methanol and subjected to analysis.

Free fatty acids were analyzed by UPLC/QTOFMS. The elution solvents were water (A) and acetonitrile/isopropyl (v/v = 80/20, B) with a flow rate of 400 μL/min. The initial gradient was 70% B and kept for 2 min, increased to 75% B in 3 min, increased to 80% in 5 min, increased to 90 in 3 min, increased to 99% in 3 min, and kept at 99% for 5 min before switching back to initial condition. The MS was operated at a positive electrospray ionization mode. One standard calibration solution with 65 free fatty acid standards at 10 different concentration levels was analyzed every five sample injections. The peak annotation and quantitation was performed by TargetLynx application manager (Waters Corp., Milford, MA, USA).

#### Metabolite profile assessment

A total of 175-μL methanol/chloroform (3:1) and internal standard solution (10 μL of p-chlorophenylalanine in water, 0.1 mg/mL) was added to 10 mg of caecal content sample, homogenized for 5 min, and centrifuged at 13,200 *g* at 4 °C for 15 min. A 200-μL aliquot of supernatant was transferred to a GC sampling vial and vacuum dried for further TMS derivatization. Methoxyamine (50 μL; 15 mg/mL in pyridine) was added to the dried sample, and the sample was maintained at 30 °C for 1.5 h. N,O-Bis(trimethylsilyl)trifluoroacetamide (50 μL; containing 1% trimethylchlorosilane) was added and the sample was kept at 70 °C for 1 h. The sample was vortexed for 10 sec prior to analysis.

Metabolite profiling was performed on GC-TOFMS. Pooled extraction quality control sample was injected every 10 sample injections. Each 1-μL aliquot of the derivatized solution was injected into the instrument. Separation was achieved on a DB-5 MS capillary column (30 m × 250 μm I.D., 0.25 μm film thickness; (5%-phenyl)-methylpolysiloxane bonded and crosslinked) with helium as the carrier gas at a constant flow rate of 1.0 mL/min. The temperature of injection, transfer interface, and ion source was set to 270, 260, and 220 °C, respectively. The GC temperature programming was set to 2 min isothermal heating at 80 °C, followed by 10 °C/min oven temperature ramps to 140 °C, 4 °C/min to 210 °C, 10 °C/min to 240 °C, and 25 °C/min to 290 °C, with a final 4.5 min maintenance at 290 °C. Electron impact ionization (–70 eV) at full scan mode (m/z 30–600) was used, with an acquisition rate of 20 spectra/s.

Spectral data analysis was performed by ChromaTOF software. Compound identification was performed using our in-house library containing over 1000 mammalian metabolite standards and online available libraries (National Institute of Standards and Technology).

### Gut microbe 16*S* rRNA sequencing

Five caecal content samples of each group were selected for microbiota 16*S* rRNA analysis. Microbial genome DNA was extracted from samples using a QIAamp DNA stool mini kit (QIAGEN, cat#51504) following the manufacturer’s protocol. Successful DNA isolation was confirmed by agarose gel electrophoresis.

The V4-V5 hypervariable regions of 16*S* rRNA gene were PCR amplified from microbial genomic DNA harvested from caecal samples and used for the remainder of the study. PCR primers flanking the V4-V5 hypervariable region of bacterial 16*S* rDNA were designed. The barcoded fusion forward primer was 520 F 5-AYTGGGYDTAAAGNG-3, and the reverse primer was 802R 5-TACNVGGGTATCTAATCC-3. The PCR conditions were as follows: one pre-denaturation cycle at 94 °C for 4 min, 25 cycles of denaturation at 94 °C for 30 s, annealing at 50 °C for 45 s, elongation at 72 °C for 30 s, and one post-elongation cycle at 72 °C for 5 min. The PCR amplicon products were separated on 0.8% agarose gels and extracted from the gels. Only PCR products without primer dimers and contaminant bands were used for sequencing by synthesis. Barcoded V4-V5 amplicons were sequenced using the pair-end method by Illumina Miseq with a six-cycle index read. Sequences with an average phred score lower than 25, ambiguous bases, homopolymer runs exceeding 6 bp, primer mismatches, or sequence lengths shorter than 100 bp were removed. Only sequences with an overlap longer than 10 bp and without any mismatch were assembled according to their overlap sequence. Reads that could not be assembled were discarded. Barcode and sequencing primers were trimmed from sequence reads [30, 36].

### Taxonomy classification

Taxon-dependent analysis was conducted using the Ribosomal Database Project classifier [37], which is a web-based program that assigns 16*S* rRNA gene sequences to phylogenetically consistent bacterial taxonomy. Bacterial operation taxonomic units (OTU) were generated using the uclust function in QIIME (http://qiime.org/scripts/pick_otus.html), and were counted for each sample to express the richness of bacterial species with an identity cutoff of 97%. The OTU abundance of each sample was generated at the species level. The mean length of all effective bacterial sequences without primers was 223 bp. Simpson indices were calculated by MOTHUR (http://www.mothur.org/) [38].

### Statistical analysis

All of the physiological, biochemical, metabolite, and microbiota data are collected from different individuals (seven or five per group) as biological replicates. Taxonomy abundance at different ranks was normalized to the summation by each sample. A phylogenetic tree was constructed based on the 16*S* sequence alignment using clearcut in MOTHUR. Unweighted UniFrac was run using the resulting tree, and PCOA was performed on the resulting matrix [39]. The linear discriminant analysis effect size method was used to compare significant differences in taxa between groups [40]. The Wilcoxon signed-rank and Wilcoxon rank-sum tests were used to compare groups of paired samples and unpaired samples, respectively, and were performed using STATA 12.0 I/C (Statacorp, College Station, TX, USA). Principal component analysis and partial least squares discriminant analysis were carried out in the Simca-P 13.0 software package (Umetrics, Umea, Sweden). Mann–Whitney U tests and Spearman correlation were performed using SPSS 13.0 software. The statistical significance *P* values were adjusted using a false discovery rate of 0.05.

## Additional files

Additional file 1: Table S1. Physiological and biochemical parameters of mice in each group. (XLSX 21 kb)

Additional file 2: Figure S1. The percentage of the number of metabolites in each type accounting for the total number. (DOC 50 kb)

Additional file 3: Table S2. Metabolome profile of caecal content in control and HFD groups at 8 weeks. (XLSX 39 kb)

Additional file 4: Table S3. Correlation of metabolite types and microbial phyla. (XLSX 16 kb)

Additional file 5: Table S4. Concentrations of bile acids in caecal content in each group. (XLSX 48 kb)

Additional file 6: Figure S2. The microbial community diversity in control and HFD groups shown by Simpson reciprocal index. (DOC 54 kb)

Additional file 7: Table S5. Microbiota at phylum levels in each group. (XLSX 12 kb)

Additional file 8: Figure S3. A linear discriminant analysis effect size cladogram representing the significantly different taxa in a tree-like structure. (DOC 249 kb)

Additional file 9: Table S6. Linear discriminant analysis effect size analysis of microbiota in control and HFD groups. (XLSX 15 kb)

Additional file 10: Table S7. Correlation of bile acids and microbiota in control and HFD groups. (XLSX 21 kb)

Additional file 11: Figure S4. The trajectories of bile acids and microbiome alteration along 56 days in control and HFD groups. (DOC 167 kb)

Additional file 12: Table S8. Time dependent alteration of bile acids and microbes in control and HFD groups. (XLSX 33 kb)

Additional file 13: Figure S5. The microbial community diversity in control, HFD, and control + BA groups shown by Simpson reciprocal index. (DOC 66 kb)

Additional file 14: Table S9. Correlation of bile acids and microbiota in control and control + BA groups. (XLSX 22 kb)

Additional file 15: Figure S6. The microbial community diversity in control, HFD, and HFD + GW4064 groups shown by Simpson reciprocal index. (DOC 72 kb)

Additional file 16: Table S10. Feed ingredients. (XLSX 10 kb)
