# Supplementary material for: Bile acid is a significant host factor shaping the gut microbiome of diet-induced obese mice
Source: BMC Biol. 2017 Dec 14;15:120. doi: 10.1186/s12915-017-0462-7 (PMC5731064; doi:10.1186/s12915-017-0462-7)
Supplement: Supplementary file 2 — The percentage of the number of metabolites in each type accounting for the total number. (DOC 50 kb) [file 12915_2017_462_MOESM2_ESM.doc]

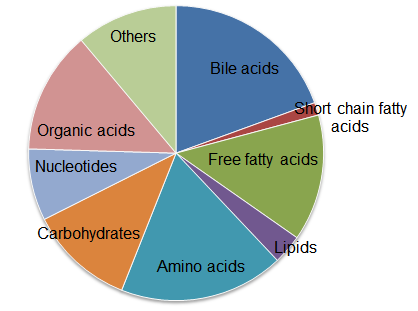


**Figure S1.** The percentage of the number of metabolites in each type accounting for the total number. A total of 211 metabolites were detected in the caecel content of mice fed with normal chow (n = 5) and high fat diet (n = 5), including bile acids, short chain fatty acids, free fatty acids, lipids, amino acids, carbohydrates, nucleotides, organic acids,.etc.. The size of each pie slice in the pie chart displays the relative proportion of each type of metabolites among all the detected metabolites.
