# Supplementary material for: Bile acid is a significant host factor shaping the gut microbiome of diet-induced obese mice
Source: BMC Biol. 2017 Dec 14;15:120. doi: 10.1186/s12915-017-0462-7 (PMC5731064; doi:10.1186/s12915-017-0462-7)
Supplement: Supplementary file 6 — The microbial community diversity in control and HFD groups shown by Simpson reciprocal index. (DOC 54 kb) [file 12915_2017_462_MOESM6_ESM.doc]

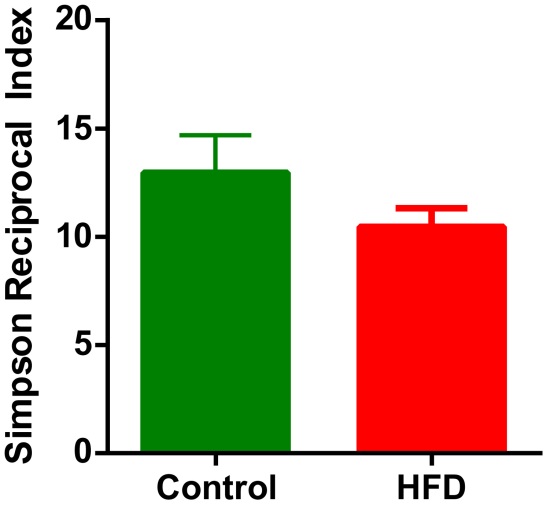


**Figure S2**. The microbial community diversity in control and HFD groups shown by Simpson reciprocal index. Data are expressed as mean ± SEM.
