# Supplementary material for: Bile acid is a significant host factor shaping the gut microbiome of diet-induced obese mice
Source: BMC Biol. 2017 Dec 14;15:120. doi: 10.1186/s12915-017-0462-7 (PMC5731064; doi:10.1186/s12915-017-0462-7)
Supplement: Supplementary file 8 — A linear discriminant analysis effect size cladogram representing the significantly different taxa in a tree-like structure. (DOC 249 kb) [file 12915_2017_462_MOESM8_ESM.doc]

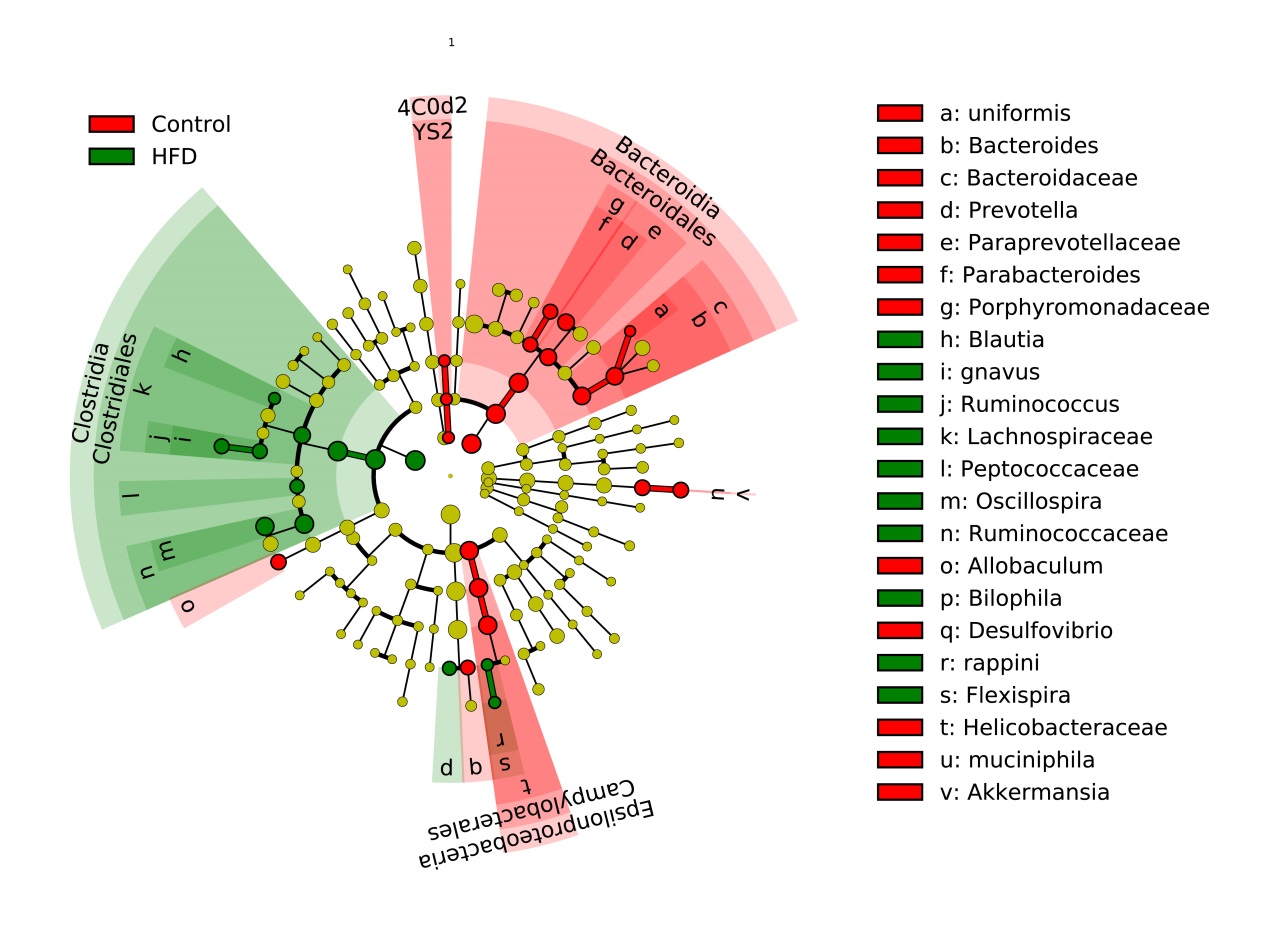


**Figure S3**. A LEfSe cladogram representing the significantly different taxa in a tree-like structure. Red indicates taxa enriched in control group, and green indicates taxa enriched in HFD group.
