# Supplementary material for: Bile acid is a significant host factor shaping the gut microbiome of diet-induced obese mice
Source: BMC Biol. 2017 Dec 14;15:120. doi: 10.1186/s12915-017-0462-7 (PMC5731064; doi:10.1186/s12915-017-0462-7)
Supplement: Supplementary file 11 — The trajectories of bile acids and microbiome alteration along 56 days in control and HFD groups. (DOC 167 kb) [file 12915_2017_462_MOESM11_ESM.doc]

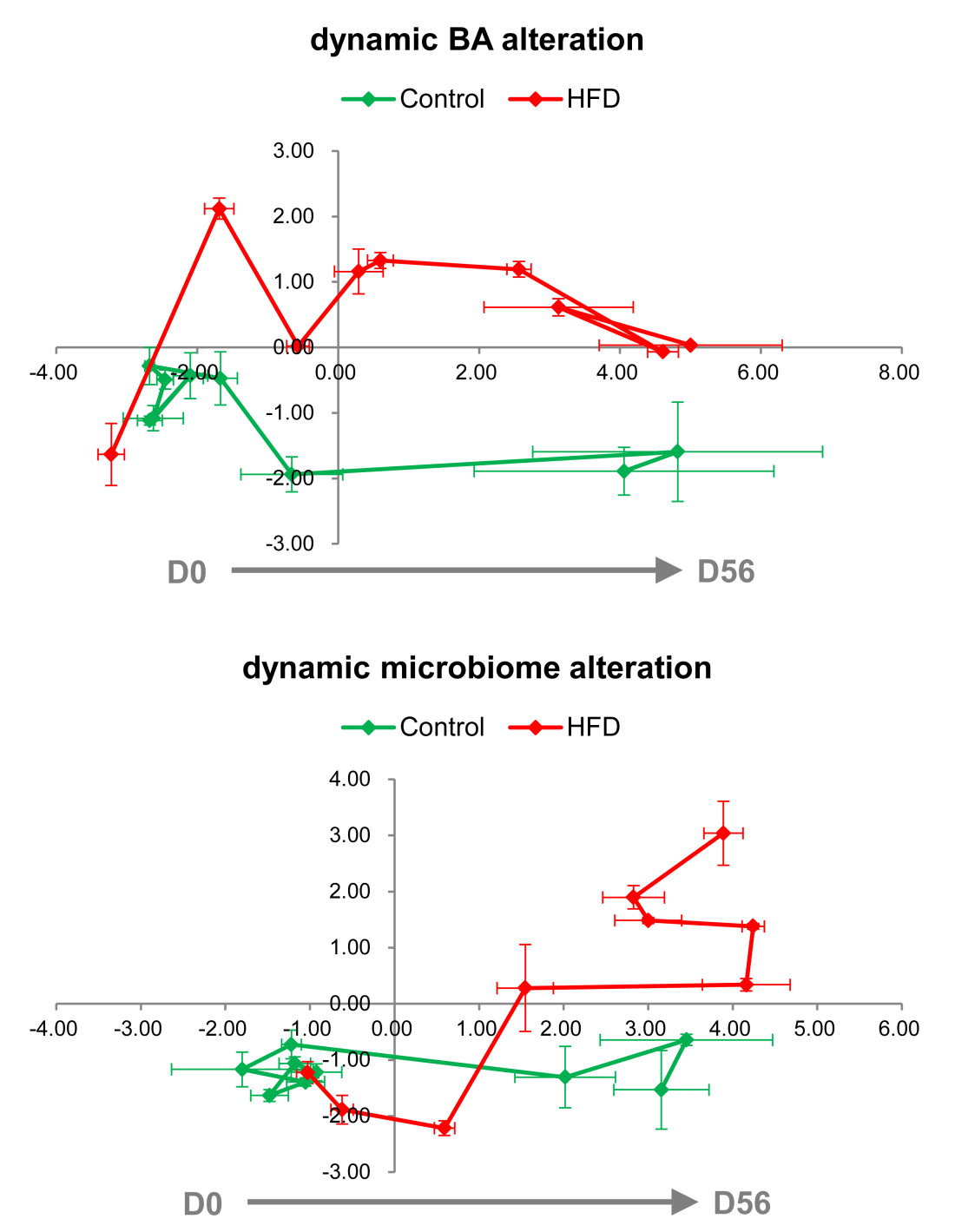


**Figure S4.** The trajectories of bile acids and microbiome alteration along 56 days in control and HFD groups. The trajectories are shown based on t values of principal component 1 (PC1) and PC2 by partial least squares discriminant analysis (PLS-DA). Data are expressed as mean ± SEM.
