# Supplementary material for: Bile acid is a significant host factor shaping the gut microbiome of diet-induced obese mice
Source: BMC Biol. 2017 Dec 14;15:120. doi: 10.1186/s12915-017-0462-7 (PMC5731064; doi:10.1186/s12915-017-0462-7)
Supplement: Supplementary file 15 — The microbial community diversity in control, HFD, and HFD + GW4064 groups shown by Simpson reciprocal index. (DOC 72 kb) [file 12915_2017_462_MOESM15_ESM.doc]

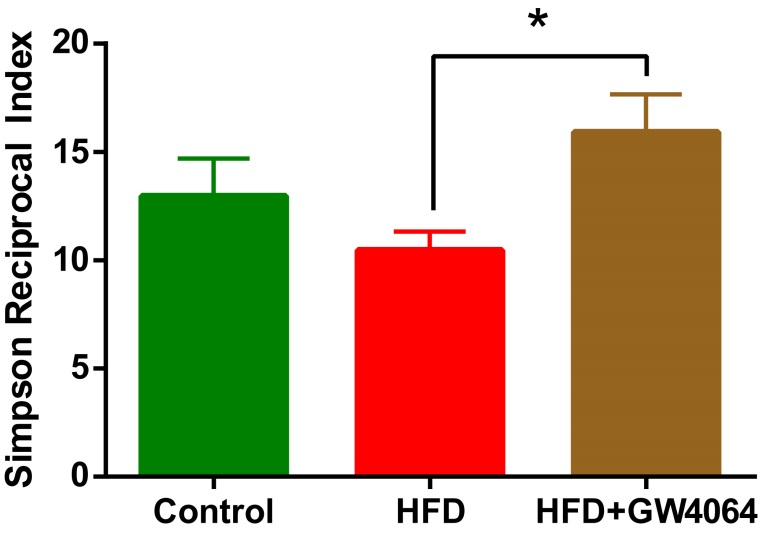


**Figure S6**. The microbial community diversity in control, HFD and HFD+GW4064 groups shown by Simpson reciprocal index. Data are expressed as mean ± SEM. The index is significantly lower in HFD groups, meaning lower α-diversity compared to that for the HFD+GW4064 group using the Mann Whitney U test. * indicates *p* < 0.05.
